# Online health information–seeking behavior by endocrinology patients

**DOI:** 10.1007/s42000-019-00159-9

**Published:** 2019-11-20

**Authors:** Angelos Kyriacou, Cathy Sherratt

**Affiliations:** 1 Postgraduate Medical Institute, Faculty of Health, Social Care & Medicine, Edge Hill University, Ormskirk, UK; 2 Department of Endocrinology, CEDM Centre of Endocrinology, Diabetes & Metabolism, Limassol, Cyprus; 3 Department of Endocrinology, Evangelismos Hospital, Paphos, Cyprus

**Keywords:** Health-related information, Patient education, Health information–seeking behavior, Doctor-patient relationships, Outpatients, Language skills

## Abstract

**Purpose:**

Given that the Internet is important for health-related information (HRI) and the fact that online health information (OHI)–seeking behavior has never been studied in endocrinology, we set out to examine how and why the Internet is utilized for HRI, the frequency of such activity, its impact, future information needs, and the effect of language.

**Methods:**

A mainly quantitative, embedded mixed-methods study was performed, employing a questionnaire survey. We included 312 patients (78.4% response rate).

**Results:**

OHI-seeking was reported by 175 patients (56.1%), especially in younger (*p* = 0.037) and more educated (*p* = 0.006) patients. OHI-seekers perceived OHI to be high-quality (135, 77.1%) but 104 (59.4%) were unaware of website certification tools. Among OHI-seekers, 63 (36.6%) reported positive behavioral changes after seeking OHI. Only 45 (25.7%) OHI-seekers discussed their gathered information with their endocrinologist. If an interactive e-learning module was available, 194/312 (62.2%) patients expressed willingness to use it, especially those reporting a need for more HRI (*p* = 0.024). Native speakers were more likely to report that OHI did not meet their information needs (*p* < 0.001).

**Conclusions:**

OHI-seeking by patients attending the endocrinology outpatients is widely practiced. The availability of OHI in the native language and e-learning modules may enhance the utility of the Internet for health information.

## Introduction

In modern times, the medical consultation process is regarded as an interaction between the clinician and the patient who agree on a shared decision-making pathway. For this to happen, clinicians need to understand patients’ ideas, concerns, and expectations, which, in turn, are affected by the sources of information that the patients have consulted prior to their encounter with the health care professional (HCP) [1]. With the rising importance of the Internet, a shift has been observed in obtaining HRI from traditional information sources to using the Internet. The emerging preference for, and dominance of, this medium among health information-seekers has been reported across different specialties [2–5]. Endocrinology has some unique characteristics as a specialty: it is a mainly outpatient, usually secondary or tertiary care, and is a specialty that intertwines with numerous chronic conditions and with preventative medicine. For endocrine patients, information about their condition is often challenging to understand, being both complex and highly scientific. There is an ongoing drive by professional societies and medical institutions to improve the provision of services in this specialty [6], and yet online health information (OHI)–seeking behavior has not been systematically investigated, and clinicians are unaware of their patients’ practise.

Estimates of how many people utilize the Internet for health information vary approximately between 30 and 80% [2, 3, 7–10], and it is worth examining the patients’ “online journey” via which they discover OHI. Many studies suggest an initial reliance on search engines to identify relevant websites [9, 11, 12]. Subsequently, patients use individual criteria to filter which websites are worth exploring [13]. Similarly, patients’ perceptions of the reliability and trustworthiness of websites is important, since this impacts on their decisions regarding their own management. Indeed, concerns have been expressed that patients often obtain inaccurate, outdated, outright false, or even potentially life-threatening online information [12, 14–16]. We need to comprehend the information needs that drive patients to utilize this medium; conversely, why is it that some patients choose not to use this seemingly widely available and easy-to-access resource?

OHI may have cognitive, emotional, behavioral, and clinical outcome dimensions. Many studies report increased knowledge and understanding of their health concerns among individuals that seek OHI [17]. Many, but not all, studies report a reduction in anxiety levels [10, 17]. Overall, there seems to be an improvement of patients’ perceived self-control and self-management skills [12, 17, 18].

Moving forward, we need to appreciate how well OHI currently meets the information needs of endocrine patients and how keen our patients are to access additional features, such as interactive e-learning modules, that could enhance their understanding of their health concern. Only then will we be able to consider how the Internet can be best “manipulated” to serve the information needs of our patients.

The overarching research questions were (a) the extent of OHI-seeking behavior; (b) how the Internet is utilized for HRI and its perceived reliability; (c) why OHI is sought prior to seeing the endocrinologist; (d) the impact of OHI-seeking behavior in general and specifically for doctor-patient relationships; (e) to assess the future information needs of participants; and (f) the correlation between language skills and OHI-seeking behavior.

## Methods

A questionnaire survey was used to investigate where, how, and why our patients seek online HRI. This employed a simple, embedded mixed methods approach, whereby quantitative and qualitative data were collected together (albeit with a greater focus on quantitative rather than qualitative data) [19]. Data from closed questions were coded, whereas qualitative data underwent thematic analysis [20] (see Appendix 1a, b).

A convenience sample was chosen from the outpatient population attending the endocrine clinic at CEDM Centre of Endocrinology, Diabetes & Metabolism, Limassol and Evangelismos Hospital, Paphos, Cyprus, between June and September 2017. This study was an academic collaboration with Edge Hill University, UK. Inclusion criteria were patients aged 18-65 years. Exclusion criteria were age > 65 or < 18 years, severe disability, severe psychiatric impairment, and terminal illness. Moreover, attendance for a non-illness-related reason (e.g., insurance check-up) and for a non-endocrine complaint (e.g., patients erroneously referred) were also excluded, as were patients who returned to the clinic but had already completed the questionnaire. During the consultation, the endocrinologist did not deviate from their usual clinical practice; patients were not specifically asked anything about their information-seeking habits. All patients who fulfilled the inclusion criteria were informed about the purpose and practicalities of the study and were invited to participate at the end of the consultation. If they wished to participate, they filled in a self-completion questionnaire, usually immediately, although a minority of participants chose to complete the questionnaire at their convenience and return it to the clinic. These questionnaires were included, provided they were returned within 1 week. The questionnaire was available in English and Greek, and patients chose questionnaires in their preferred language.

Ethical approval was granted by Edge Hill University, UK (reference FOHS176). The Bioethics Committee of Cyprus was also formally consulted and confirmed that the study required no additional approval. Permission for the study was granted by the Evangelismos Hospital management.

Quantitative variables are presented using descriptive statistics, while categorical variables are presented as absolute and relative (%) frequencies. To investigate associations between categorical variables, the chi-square test and Fisher’s exact test (*χ*^2^) were used. For correlation between interval (quantitative) variables, Pearson’s *r* (Pr) test was undertaken. Ordinal regression analysis was performed to investigate the relationship between the language preferred by the patients for the completion of the questionnaire and the extent to which the patients perceived that the Internet covered their information needs. Bonferroni correction was applied post hoc as required for multiple variable calculations. SPSS v20 (IBM Corp. 2011) was used for statistical analysis. A two-sided *P* < 0.05 was considered statistically significant.

## Results

### Demographics

Of the 398 patients who fulfilled the inclusion criteria, 312 agreed to participate, yielding a response rate of 78.4%. Patient demographics are displayed in Table 1. Patients were also asked to rate how satisfied they were with the information they received as part of their consultation with their doctor, with 181 (58%), 107 (34.3%), 11 (3.5%), 2 (0.6%), and 7 (2.2%) patients reporting that they were very satisfied, satisfied, neither satisfied nor unsatisfied, unsatisfied, or very unsatisfied, respectively.

**Table 1:** Patient demographics

Parameter		Results, *n* (%)
Age (years)	18-35	106 (34)
	36-49	85 (27.2)
	50-64	119 (38.1)
	Missing values	2 (0.6)
Gender	Males	61 (19.6)
	Females^a^	251 (80.4)
Education level	Middle school or lower	7 (2.2)
	High school	107 (34.3)
	University	127 (40.7)
	Postgraduate	66 (21.2)
	Missing values	5 (1.6)
Marital status	Single	58 (18.6)
	Married	200 (64.1)
	Divorced	27 (8.7)
	Widowed	7 (2.2)
	Cohabiting	13 (4.2)
	Missing values	7 (2.2)
Home location	Urban	235 (75.3)
	Rural	66 (21.2)
	Missing values	11 (3.5)
Annual household income^b^(in Euros and including the partner’s income)	0–10,000	55 (17.6)
	10,001–20,000	78 (25)
	20,001–30,000	35 (11.2)
	30,001–60,000	51 (16.3)
	> 60,000	30 (9.6)
	Missing values	63 (20.2)
Type of appointment	First (new patient)	113 (36.2)
	Follow-up	196 (62.8)
	Missing values	3 (1)
Source of information utilized^b^	Family	80 (25.6)
	Friends	69 (22.1)
	Neighbors	2 (0.6)
	Work colleagues	28 (9)
	TV	13 (4.2)
	Radio	2 (0.6)
	Internet	175 (56.1)
	Newspapers	4 (1.3)
	Magazines	10 (3.2)
	Books	25 (8)
	Other	25 (8)

^a^The comparative proportion of females in these clinics is approximately 75%

^b^Answers were not mutually exclusive

### Uptake and extent of online health information-seeking

Among the whole study population, 224 (71.8%) patients reported health information–seeking behavior prior to their appointment. The Internet was by far the commonest source of information (175, 56.1% of the whole population and 78.1% of health information-seeker subgroup, respectively). Indeed, the frequency of Internet use for health information purposes matched that of all other (traditional) sources of information combined (178, 57% of the whole study population) (Table 1).

On interrogating demographics, only younger patients and the more educated were significantly more likely to exhibit OHI-seeking (Table 2).

**Table 2:** Relationship of demographic parameters with online health information (OHI)–seeking status

Demographics^a^		OHI-seeking		Bonferroni corrected *p* value^bc^
		Yes, *n* (%)	No, *n* (%)	
Age group (years)	18–35	68 (64.2)	38 (35.8)	*0.007*
	36–49	55 (64.7)	30 (35.3)	
	50–64	50 (42)	69 (58)	
Gender	Male	28 (45.9)	33 (54.1)	0.525
	Female	147 (58.6)	104 (41.4)	
Education status	Up to middle school	0 (0)	7 (100)	*< 0.001*
	Up to high school	42 (39.3)	65 (60.7)	
	University	80 (63)	47 (37)	
	Postgraduate	49 (74.2)	17 (25.8)	
Marital status	Single/widowed/divorced	53 (57.6)	39 (42.4)	> 0.999
	Married/co-habiting	117 (54.9)	96 (45.1)	
Location	Town	133 (56.6)	102 (43.4)	> 0.999
	Rural	35 (53)	31 (47)	
Annual household income (in Euros)	0–10000	30 (54.5)	25 (45.5)	0.532
	10,001–20,000	42 (53.8)	36 (46.2)	
	20,001–30,000	24 (68.6)	11 (31.4)	
	30,001–60,000	28 (54.9)	23 (45.1)	
	> 60000	24 (80)	6 (20)	
Appointment type	New	72 (63.7)	41 (36.3)	0.301
	Follow-up	101 (51.5)	95 (48.5)	

^a^Missing values as reported in Table 1

^b^Chi-square test or Fisher’s exact test was used as necessary for these calculations

^c^Italicised values are statistically significant at the *p*<0.05 level

OHI-seekers reported spending a mean of 85.4 min (SD 170.6 min) in the preceding month searching the Internet specifically for HRI for the health concern(s) that brought them to see the doctor. When asked how frequently they had gone online in the past month for HRI-seeking about their health concern(s), on average, they surfed the web 4.6 (6.8) times, thereby spending, on average, 18.6 min per OHI session. The number of times they logged in online was strongly correlated with time spent seeking OHI (*r* = 0.566, *p* < 0.001, Pr).

### How the Internet is utilized for health information and perceived reliability

The majority of OHI-seekers (134, 76.6%) collected their web-based information in preparation for the consultation via search engines (e.g., Google, Yahoo, and Yandex). Other ways of conducting their online search were “directly looked at specific websites” (21, 12%), “patient forums” (2, 1.1%), “Facebook” (2, 1.1%), and “others” (3, 1.7%) (missing values 13). Patients reported that the main criterion for choosing a specific website was whether it appeared first in their search engine results (71, 40.6%). Alternatively, they chose websites because they belonged to health institutions or services (38, 21.7%), academic institutions (24, 13.7%), societies for a specific illness (e.g., Diabetes UK) (17, 9.7%), Wikipedia (6, 3.4%), or “other” criteria (6, 3.4%) (missing 13).

**Fig. 1:**
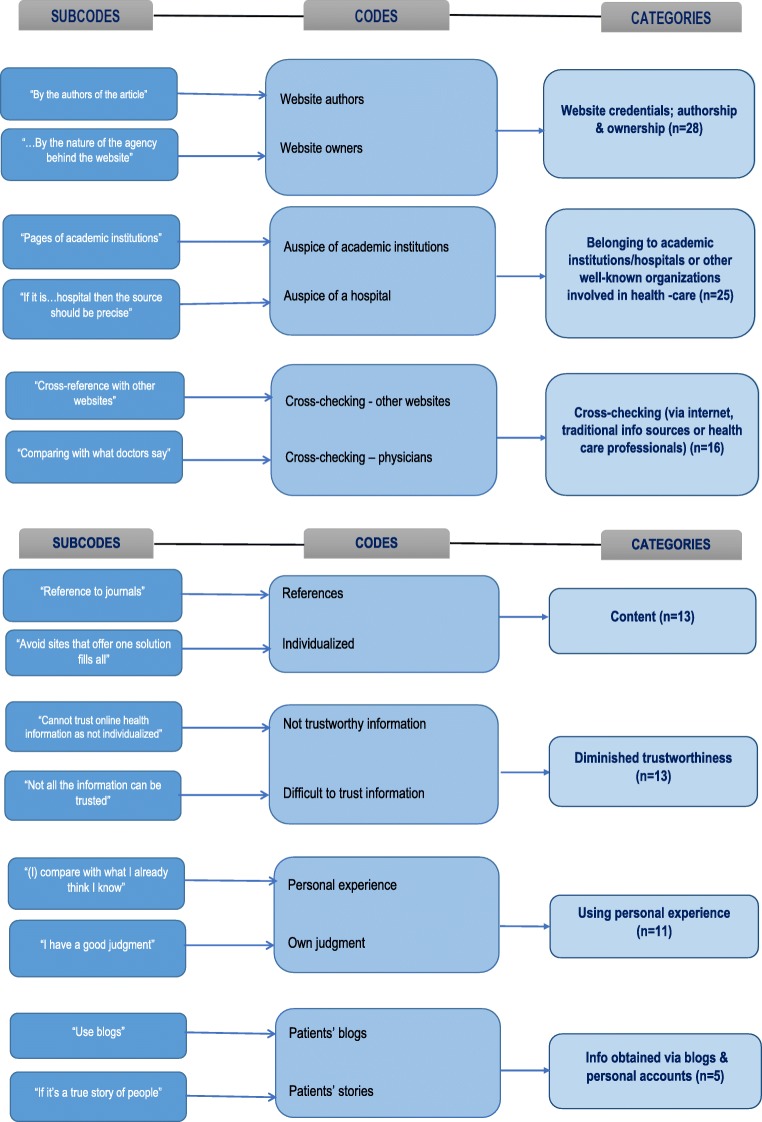
How to check whether a website has trustworthy information

Further probing into patients’ perceptions of trustworthiness was via the question: “How do you determine whether a website has trustworthy information or not?” Subsequent thematic analysis revealed that the three most frequent explanations offered by patients were (in decreasing frequency) belongs to an academic institution, belongs to a health/medical institution or authority, and if the website has been certified by their physician (Fig. 1).

When asked to rate the quality of OHI, roughly three quarters of patients reported it was either “good” or “very good,” while about half thought of OHI as “reliable” or “very reliable” (Table 4). The reporting of good or very good quality increased as education level increased (31, 73.8% vs. 60, 76% vs. 42, 85.7% for high school, university, and postgraduate education, respectively, *p* = 0.047, *χ*^2^, missing 5), and the same was true for perceived reliability of OHI (18, 45% vs. 33, 42.3% vs. 33, 70.8% for high school, university, and postgraduate education, respectively, *p* = 0.054, *χ*^2^, missing 9). Forty patients (22.9%) perceived that the quality of the information they received from their doctor was better or much better than that received from OHI, 30 (17.1%) felt it was worse or much worse than OHI, but most reported no preference (99, 56.6%; missing 6). Finally, when asked whether they are aware of how to identify websites whose HRI has been certified in terms of its reliability (e.g., by the Health On the Net (HON) Foundation), most participants reported a lack of awareness (104, 59.4%).

### Why patients go online for health information prior to their endocrine outpatient’s review: why not?

The commonest reason for seeking OHI in relation to their health concern prior to their appointment was “to gather general information” (Table 3).

**Table 3:** Reasons for using and not using the Internet for health-information gathering

Reason for using the Internet^a^	*n* (%)^b^	Reason for not using the Internet^a^	*n* (%)^c^
To gather general information	90 (51.4)	No point if visiting the doctor	58 (42.3)
To be active in own health care	20 (11.4)	No Internet access	17 (12.4)
To research treatments or medication options	16 (9.1)	Never thought of it	16 (11.7)
To self-diagnose	14 (8)	The Internet is unreliable	12 (8.8)
To identify people with similar experiences	4 (2.3)	The Internet is confusing	9 (6.6)
To find a doctor	2 (1.1)	No time	6 (4.4)
		Not sure where to look for online health information	4 (2.9)
Other	6 (3.4)	Other	9 (6.6)

^a^Answers were mutually exclusive

^b^Missing values 23 (13.1%)

^c^Missing values 6 (4.4%)

**Fig. 2:**
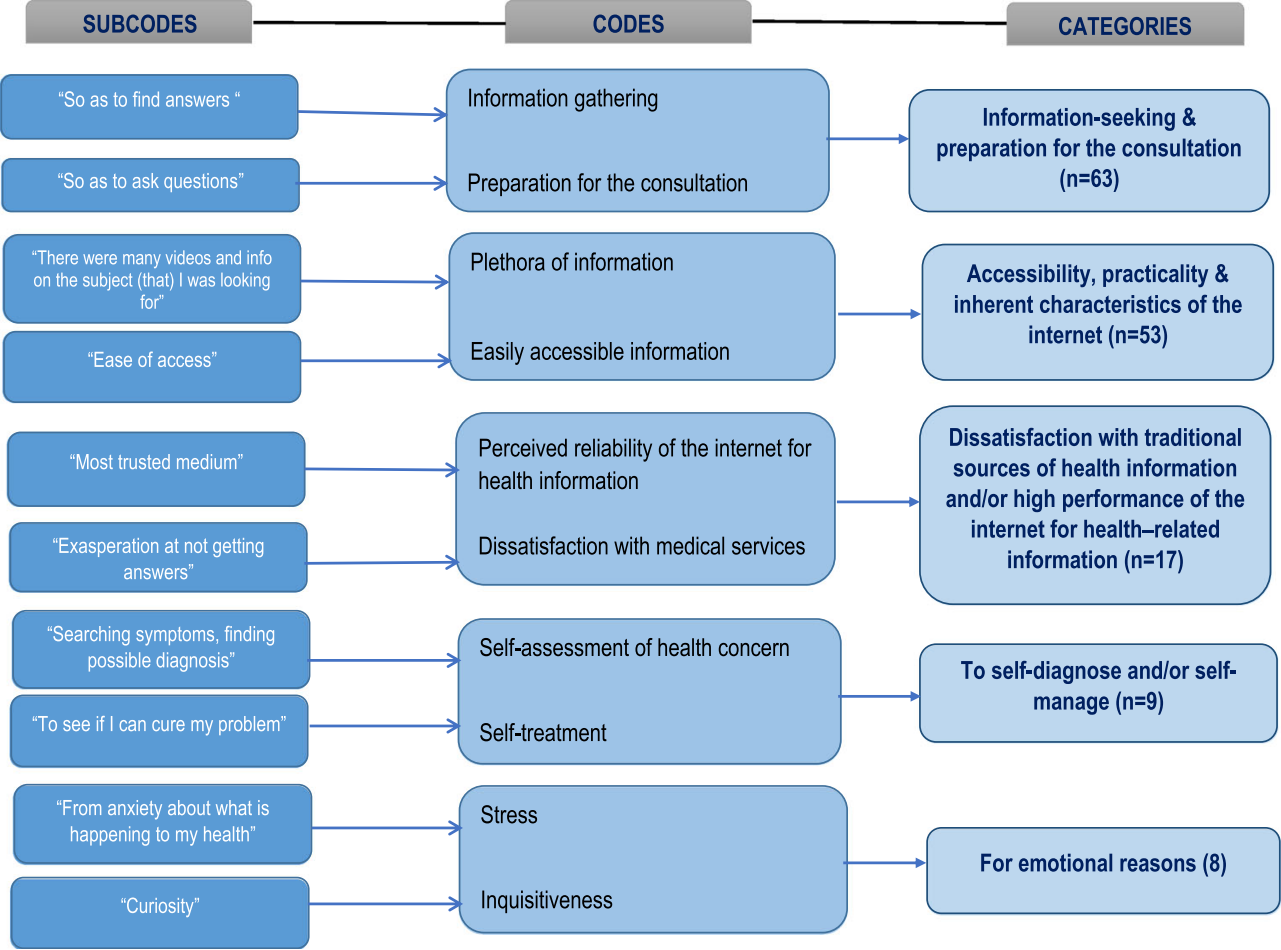
Why online health information was sought prior to the consultation

Further probing asked: “If you made use of the Internet for information gathering prior to your consultation today, why did you use this medium?” Thematic analysis revealed that the three most frequent explanations offered by patients were (in decreasing frequency): information gathering, ease of access, and because the Internet offers plenty of information (Fig. 2).

On the other hand, the commonest reason cited by non-OHI-seekers for not checking OHI was that they felt it was pointless before seeing the doctor (Table 3).

### Impact of online health information

#### Emotional and behavioral impact

Overall, more OHI-seekers reported feeling better or much better (53, 30.3%) rather than worse or much worse (14, 8%), albeit most patients reported feeling “neither better nor worse” after “seeking and finding” OHI (106, 60.6%; missing 2). Conversely, slightly more patients reported that their anxiety levels increased or markedly increased (44, 25.1%) following their online search for health information, but again, most participants remained neutral (92, 52.6%; missing 4). Over one third (63, 36.6%) of participants reported that their behavior changed after seeking OHI (e.g., by taking better care of themselves or being more compliant with taking medication), while 93 (53.1%) answered negatively; missing 18.

**Fig. 3:**
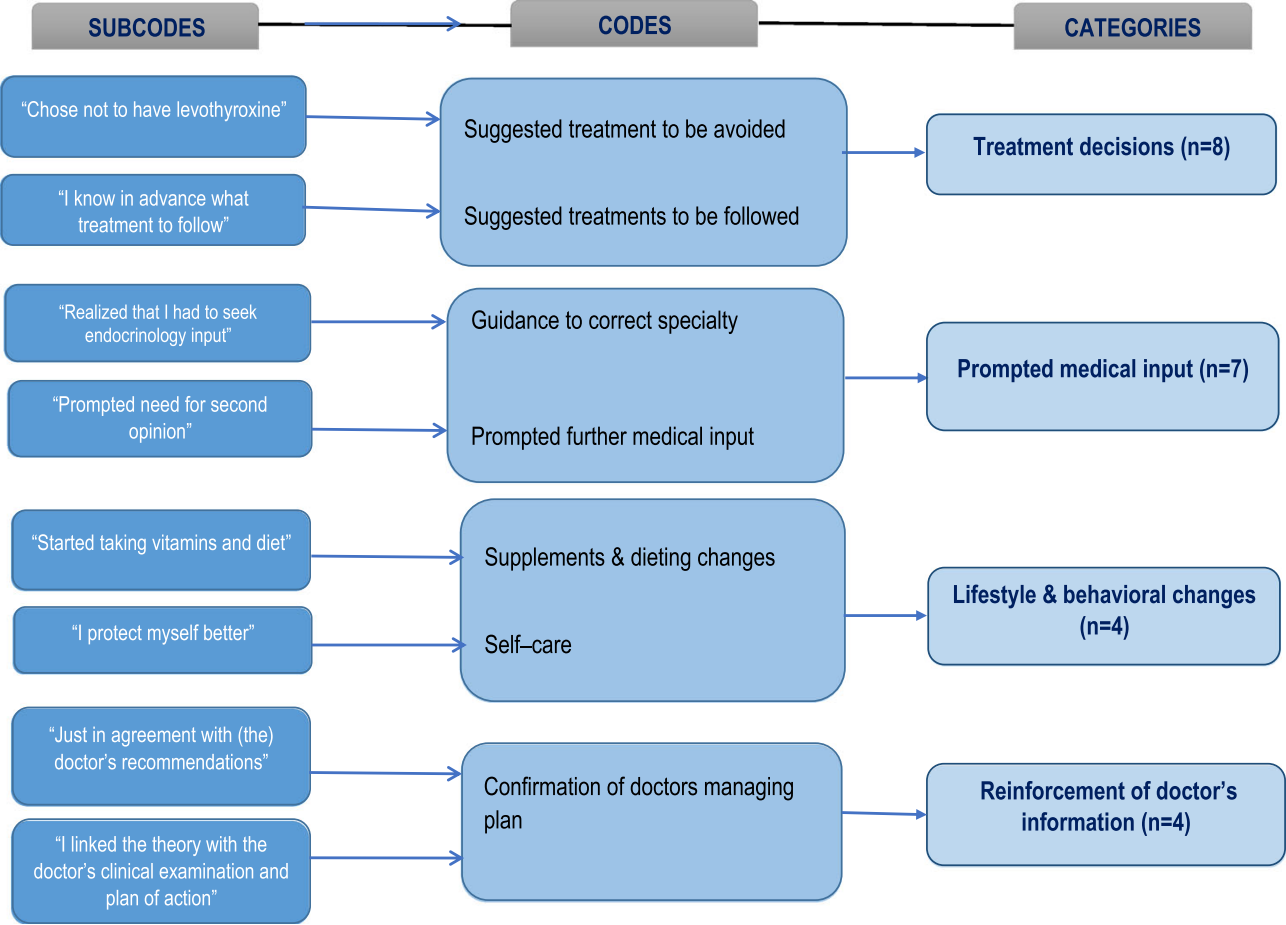
Influence of online health information on the management plan

Further probing regarding the influence of OHI on their management asked*:* “Has the information you obtained from the Internet influenced your decisions concerning your management plan in any way (for example, what investigations and/or therapies you are going to have)?” The 27 patients who answered this question positively (15.4% of OHI-seekers) were then asked to explain how it affected their management. The three most frequent explanations offered by patients were (in decreasing frequency) “better treatment choices”, reinforcement of doctor’s information, and that the information gathered prompted their visit to the doctor (Fig. 3).

#### Impact on doctor-patient relationships and satisfaction with the consultation

Only 45 (25.7%) OHI-seekers mentioned the online information during their consultation with the doctor and 117 (66.9%) did not (missing 13). The reasons why OHI was not mentioned were that the “doctor was thorough” (78, 66.7%), that they “forgot to mention it” (9, 7.7%), that “information on the Internet was not trustworthy” (6, 5.1%), or that they “did not want to appear to challenge the doctor” (4, 3.4%). Only two patients cited lack of time and one patient reported being ashamed to mention it (“other” option chosen by 9 (7.7%); missing values 8).

### Assessment of the degree of fulfillment of information needs by the Internet

Firstly, OHI-seekers were asked how well existing Internet information meets their health information needs; 80 (45.7%) answered “neither well nor poorly,” 55 (31.4%) and 2 (1.1%) answered “poorly” or “very poorly,” respectively; only 28 (16%) and 4 (2.3%) answered that their needs were “well” or “very well” met, respectively (missing 6). Education level did not influence how well OHI met patients’ health information needs (*p* = 0.338, *χ*^2^). Secondly, patients from the whole study population were asked whether they needed extra information in relation to the health concern that brought them to see the doctor, with about one third answering positively (109, 34.9%) (missing 3). Among these 109 patients, the preferred format for the additional information was face-to-face meetings with the doctor (82, 75.2%), followed by electronic (14, 12.8%), leaflets (4, 3.7%), or “other” (6, 5.5%) (missing 3). Thirdly, patients from the whole study population were asked whether they would be interested in an interactive e-learning module (software using text, pictures, videos, and presentations, and the ability to interact with the information), if it existed, for their endocrine condition, with the majority (194, 62.2%) reporting an intention or willingness to use such an e-learning module (missing 8). Existing OHI-seekers were significantly more likely to want to utilize a potential interactive e-learning module (130 (74.3%) among OHI-seekers vs. 64 (46.7%) among non-OHI-seekers, *p* < 0.001, *χ*^2^). Similarly, those who expressed a wish for more information regarding their health concern were significantly more likely to be interested in an e-learning module, 76 (69.7%) of seekers of extra information vs. 118 (59%) of non-seekers (*p* = 0.024, *χ*^2^).

### Language skills and OHI-seeking behavior

Most patients (256, 82.1%) completed the questionnaire in Greek (“GR-fillers”) and the rest in English (“EN-fillers”), with the latter subgroup being significantly more likely to report that their information needs were well met by the Internet (Table 4). However, only about a quarter of patients performed their online search solely in Greek, with a third searching in both Greek and English or English alone. The language used to complete the questionnaires did relate to the language in which the Internet search was performed (*p* < 0.001, *χ*^2^). Patients who performed their online search in English were significantly more likely to report that OHI met their health information needs well or very well (15 (26.8%) for English, vs. 9 (14.8%) for both English and Greek, vs. 1 (2.3%) for Greek language alone, *p* < 0.001, *χ*^2^); the result remained significant when adjusted for age and education level by ordinal regression analysis (*p* = 0.033, Nigelkerke *R*^2^ = 7.8%). The preferred language for any additional information was Greek (63, 57.8%), followed by English (23, 21.1%) and dual Greek/English (22, 20.2%).

**Table 4:** Preferred language for questionnaire completion and online health information-seeking behavior

Attribute	“GR-fillers”, n (%)^c^	“EN-fillers”, n (%)^c^	Bonferroni-corrected *p* value^b^
OHI status^a^	141 (55.1)	34 (60.7)	> 0.999
Feeling better or much better after seeking OHI	14 (10.2)	19 (57.6)	*< 0.001*
Anxiety levels reduced or markedly reduced post-OHI	26 (18.8)	10 (29.4)	> 0.999
Rating of quality of OHI as good or very good	109 (77.9)	27 (79.4)	> 0.999
Perception of OHI as being reliable or very reliable	71 (52.6)	15 (44.1)	> 0.999
OHI meeting patients’ information needs well or very well	14 (10.2)	19 (57.6)	*< 0.001*

Italicised values are statistically significant at the *p*<0.05 level

^a^Taken out of the whole study population, whereas the rest of the table refers solely to the OHI-seekers.

^b^Chi-square test or Fisher’s exact test were used as necessary for these calculations.

^c^“*EN-fillers*” patients who completed the English version of the questionnaire, “GR-fillers” patients who completed the Greek version of the questionnaire, *OHI* online health information

## Discussion

### Uptake and extent of online health information-seeking

In this first study of OHI-seeking behavior among endocrine patients, we have shown that 56% of such patients consult the Internet for health information prior to consulting their endocrinologist. Most importantly, the Internet is now established as the dominant medium for health information-seekers, with its uptake being similar to that of all other information sources combined. Younger and more educated patients were significantly more likely to search for OHI. Increased OHI use by the more educated may imply that OHI is more accessible to already advantaged population subgroups, which has significant public health implications.

### How the Internet is utilized for health information and perceived reliability

Consistently with previous studies [9, 11, 12], three quarters of these patients commenced their online search for HRI using a search engine. Thereafter, they chose to gather more information from a specific website based on the first few options provided by their search engine (40.6%) or, alternatively, they selected sites belonging to health or academic institutions or societies with an interest in a specific illness (45.1%). Search engines use complex algorithms to decide which sites appear first; hence, many patients may be accessing less informative and/or reliable websites. Furthermore, sponsored or advertised websites commonly appear first on search engines, with all the inherent conflicts of interest. Conversely, our open question on website reliability revealed that a significant majority of patients are well aware of how to identify trustworthy websites, with most attention drawn to areas of ownership, authorship, and website content (Fig 1). Some patients stated that they judged the reliability of a website by cross-checking with other websites or with information received from HCPs, which resonates with earlier findings [21]. Previous research has also emphasized that easy to locate and comprehend OHI relates to increased trust in such information [22]. Finally, some rely on using personal experience to judge the reliability of a website and others consider blogs and personal accounts as the most trustworthy information. Such sources or ways of judging OHI are very subjective and are more likely to lead to the wrong conclusions. This means that there is room for improved e-health literacy skills among our patients, a conclusion also drawn from the fact that none of our participants mentioned any online health accreditation tools.

About half the patients felt the Internet gives reliable health information, with three quarters expressing an opinion that OHI was good or excellent quality. There was a trend of borderline statistical significance, with the more educated patients considering the OHI they obtain as being better quality and more reliable, which may reflect greater sophistication in their web searching.

Overall, this indicates that our patients regard the Internet as a reliable information medium and the majority can appreciate which are the essential elements of trustworthy websites. However, substantial work is still needed to educate all patients that reliance on the top options from search engines is neither a sufficient nor an efficient way of searching for OHI. We also need to boost patients’ confidence in the standard of OHI and how to check for HONcode certification or similar accreditation.

### Why patients go online for health information prior to their endocrine outpatient’s review: why not?

The most common reason cited for OHI-seeking was gathering more information related to the patient’s health concern. This was confirmed by open questioning, which provided the additional insight that patients simply seek OHI because it is conveniently easy to access and there is plenty of it! This is consistent with previous studies [2, 10]. Indeed, if there is the right (infra)structure to guide patients to the best information channels before their consultation, then, this may positively impact on the quality and outcomes of the consultation. Unlike the concerns often expressed by HCPs, only a small minority (8%) of our patients used this medium to self-diagnose, a finding not dissimilar to other studies [2, 23]. Individuals also consulted the Internet to confirm, supplement, and complement the information gathered during the consultation [10, 23] and, on occasion, because they were dissatisfied with the quality or quantity of the information or the degree of empathy received from their doctor [24, 25] or to tackle health-related anxiety.

On the other hand, 44% of health information-seekers did not utilize the Internet for HRI. The most commonly reported reason for not doing so was that they considered it pointless since they were due to see their physician. Our results differ from a survey performed in 2008 in primary care, which reported that the main reason for not seeking OHI was lack of Internet access (46.2%) and familiarity (15.4%) [26]. The difference from our study likely represents changing attitudes over the past decade, characterized by by increased Internet access and familiarity. Future public health interventions to increase OHI access may target this group.

### Impact of online health information

#### Emotional and behavioral impacts

Over a third of our study participants perceived that their behavior improved following OHI-seeking. Increased compliance, self-care, and self-management skills have previously been reported, namely, an enhanced sense of control and coping that comes with increased knowledge on their health condition [17, 18, 26, 27]. However, the majority of our patients reported feeling no better or worse, whereas both positive and negative emotional outcomes (increased or reduced anxiety) were previously reported post-OHI-seeking, with a predominance of positive outcomes, especially if patients managed to obtain the information they desired initially [17, 18, 21, 26, 28]. We can speculate that such emotional responses are complex and dependent on a variety of parameters, e.g., e-health literacy skills, level of insight, idiosyncratic and cultural factors, underlying health concern(s) per se, and website-specific factors. It is also conceivable that there is intra-individual variability in emotional responses, depending on context, time frame, and other factors; therefore, this topic warrants further research.

Although only a small portion (15%) of OHI-seekers felt that their gathered OHI influenced their management plan, subsequent qualitative analysis indicated some very encouraging revelations, e.g., that the information obtained was used in the balancing process of what management plan to follow prompted the patient to see the endocrinologist, encouraged a healthier lifestyle, improved self-care abilities, or reinforced the information provided by the doctor. Nevertheless, the majority of patients did not report an impact on their management plan. We did not explore further why that was so, but possible explanations were that they felt that the main force behind their management decisions was the information obtained through their doctor or that the patients consider OHI as being more of supplementary, background information or that there is a subconscious untrustworthiness regarding this information medium.

#### Impact on doctor-patient relationships and satisfaction with the consultation

Consistently with previous studies [2, 29], only about a quarter of OHI-seekers discussed their findings with the doctor. In our study, the main reason reported by patients for not discussing OHI was that they felt the doctor was thorough, which possibly indicates an alignment between the OHI and information provided by the doctor. However, this contrasts with the study by Hay et al. (2008) where the reasons cited by rheumatology outpatients for not communicating their OHI to the doctor was that they “did not want to challenge the physician” (12%) or “thought of it as background information only” (10.6%), with only 5% reporting that it was “unnecessary because physician was thorough” [2]. Culture, health-care setting, disease-specific factors, or date may explain these differences. Concerningly, patients who struggle to comprehend OHI appear to be similarly hesitant to discuss it with their HCPs [30].

Overall, we can conclude that OHI does not adversely affect the doctor-patient relationship and that the doctor is still rated as the most authoritative and trustworthy source of information because (i) the majority that did seek OHI did not even discuss their OHI since they felt the doctor was thorough, (ii) the great majority of our patients did not (mis)use the Internet to self-diagnose, (iii) the patients’ satisfaction rates with the doctors’ consultation were high regardless of OHI-seeking status, and (iv) face-to-face consultation with the doctor was by far the preferred information mode for those who sought further information. Similarly, other studies reported that doctors are still regarded as the principal and most reliable source of health information and support [9, 13, 26] and that trust in OHI and doctor-received information are not mutually exclusive [22]. Taken together, these findings can reassure clinicians that there is nothing to be feared by patients seeking OHI.

### Assessment of the degree of fulfillment of information needs by the Internet

Our study has shown that the majority of endocrine patients do not feel that their information needs are met by currently available web-based information. As indicated earlier, a third of these patients still need additional information post-consultation. Although the majority of these patients indicated further face-to-face contact with their doctor as their preferred source of information, given financial and time constraints, it is worth exploring if, and how, web-based resources could be used to meet at least some of these needs. Indeed, when specifically asked whether they would be willing to utilize an interactive e-learning module, about two thirds of our participants responded positively. Although existing OHI-seekers and those who sought additional information were significantly more likely to want to utilize such a module, interestingly, approximately half of non-OHI-seekers and non-seekers of extra information also expressed an intention to use it.

### Language skills and OHI-seeking behavior

Our language results are in line with the expected language skills of Cypriots, the majority of whom can speak English, although 80.9% of them report Greek as their first language [31]. A novel finding of this study is the unmet needs of native speakers when it comes to OHI; Greek-speakers were more likely to be dissatisfied with OHI and indeed the majority of participants preferred any additional OHI to be provided in Greek. People who speak English as a second language may find it more challenging or intimidating to read complex HRI in English, which is the dominant language of the Internet. An earlier study of approximately 1000 American Indians and Alaska Natives identified a lack of culturally appropriate OHI websites [32]. Consequently, these findings are of interest to anyone who manages patients whose first language is not English. These results should also raise concern among public health institutions that there is a demand for more health information to be available in local language(s).

### Strengths and limitations

This study has collected data on a large outpatient population, presenting with a variety of endocrine complaints in two different sites. A well-designed questionnaire was employed to answer our research questions, after reviewing the existing literature [2–4, 7–13, 23, 24, 26, 29, 33]. A high response rate was achieved. We deliberately refrained from doing an online survey of OHI-users (e.g., users of specific health websites) because that would introduce selection bias and make it more difficult to identify our target population. Moreover, the use of mixed methods research enriched the study and gave insight into our patients’ perspectives.

Nevertheless, our study has some limitations. The cross-sectional study design means that causality cannot be assumed. External validity (generalizability) cannot be assumed given that convenience sampling was employed and also because it is possible that our results could be dependent on local cultural characteristics and local information technology and e-health literacy. Although all efforts were made to design an appropriate questionnaire, this tool has not been validated per se. To our knowledge, there is no validated and universally accepted tool to study OHI-seeking behavior. Similarly, this raises questions regarding reliability (consistency of measures); the stability of the measure has not been tested by a formal pilot study, albeit prior to implementation, the questions were tested by colleagues including lay people. However, evidence for high internal reliability comes from the fact that some questions which deliberately probed similar topics gave similar results and that the quantitative and qualitative results of the study were congruent with each other, albeit they also provided some welcome supplementary information. For multiple variable analyses, we applied the Bonferroni correction to reduce familywise error rate, but we acknowledge the increased risk of type 2 errors and the lack of consensus with this approach [34, 35].

## Conclusion

The majority of endocrinology patients practice OHI-seeking prior to their appointments, especially the younger and more educated patients. Most patients perceive OHI as being good quality and reliable, and they appear to have some awareness of how to identify websites with trustworthy information. Nevertheless, about 40% rely on the websites provided as first options by their search engine. Most patients do not discuss their gathered OHI with their endocrinologist. There is a striking relationship in that Greek-speakers were less satisfied with OHI and demanded more OHI in their native language. If an interactive e-learning module was available, about three quarters of OHI-seekers and up to half of non-OHI-seekers would be keen to utilize it.

These findings will inform endocrinologists regarding the OHI habits of their patients. Our data should not only reassure endocrinologists that there is nothing to be feared by patients seeking OHI but should also provide an evidence-base to encourage patients to discuss any gathered OHI with them. Going a step further, we would also argue that endocrinologists need to become “Internet prescribers” and guide their patients regarding how and where to search and identify credible OHI. Moreover, our results make an argument for more online health resources to be available in the native language of users.
